# Detection of the clarithromycin resistance of *Helicobacter pylori* in gastric mucosa by the amplification refractory mutation system combined with quantitative real‐time PCR

**DOI:** 10.1002/cam4.1986

**Published:** 2019-03-12

**Authors:** Xiao‐Yan Zhang, Wei‐Xiang Shen, Chun‐Feng Chen, Hai‐Hui Sheng, Hong Cheng, Jiang Li, Fulian Hu, Da‐Ru Lu, Heng‐Jun Gao

**Affiliations:** ^1^ State Key Laboratory of Genetic Engineering, School of Life Sciences Fudan University Shanghai China; ^2^ National Engineering Center for Biochip at Shanghai Shanghai China; ^3^ Department of Gastroenterology Peking University First Hospital Beijing China; ^4^ Tongji Institute of Digestive Disease & Department of Gastroenterology Tongji University Shanghai China

**Keywords:** ARMS‐PCR, *Helicobacter pylori*, mutation, resistance

## Abstract

The goal of this study was to evaluate the feasibility of detecting Helicobacter pylori clarithromycin resistance in gastric mucosa using the amplification refractory mutation system combined with quantitative real‐time PCR (ARMS‐PCR). Gastric mucosal specimens (150) were collected from patients who were unsuccessfully treated for H. pylor*i eradication. *Each specimen was divided into 2 samples. One sample was used to extract genomic DNA and detect any gene mutations of H*.* pylori produced by ARMS‐ PCR. Sequencing was used to assess the accuracy of this method. The other sample was used to culture *H. pylori*. The E‐test minimum inhibitory concentration (MIC) was used to assess clarithromycin resistance. The results were compared with a paired chi‐square test to validate the coincidence rate among the 3 methods. The coincidence rate between the sequencing and ARMS‐PCR results was 98.7%, thus verifying the accuracy of ARMS‐PCR. E‐tests detected 144 clarithromycin resistance cases, including 45 sensitivity cases; the resistance rate was 70%. The coincidence rate between the results of the E‐test and ARMS‐PCR was 97.1%, and no significant difference between the 2 methods was observed. ARMS‐PCR is a simple and fast method that has high sensitivity and specificity and can be used to detect the clarithromycin resistance of *H. pylori* in gastric mucosa. ARMS‐PCR is expected to be used to study drug resistance mechanisms and use in assays of individual therapies for *H. pylori eradication*.

## 1 INTRODUCTION

The antibiotic resistance of Helicobacter pylori is the primary reason for the failure of *H. pylori* eradication therapy in clinical practice.^1^, ^2^, ^3^ Therefore, the early detection of *H. pylori* resistance will help assist medical professionals in selecting antibiotics, improving the success ratio of treatment and avoiding antibiotic abuse. The traditional, and current, clinical method for *H. pylori* resistance detection is a drug sensitivity test. Because the drug sensitivity test necessitates the in vitro culture of *H. pylori*, which requires harsh bacterial culture conditions and a long time period, promoting the use of resistance detection in the clinic is difficult. Recently, researchers have studied the molecular mechanism of *H. pylori* resistance and have reported multiple bacterial resistance mutations for commonly used antibiotics. These molecular tests are both simpler and quicker than the traditional phenotype detection method because they require no in vitro bacterial culture.

Many studies have shown that the clarithromycin resistance mechanism of *H. pylori* is associated with mutations on the 23S ribosomal RNA (rRNA) V domain. These mutations can cause local damage to the polypeptide transferase and reduce the usefulness of drug combinations, which results in *H. pylori *resistance to macrolide antibiotics (eg, clarithromycin).^4^, ^5^ These mutations include A2142G, A2142C, A2143G, A2143C, T2717C, T2182C, A2144T, C2245T, and T2289C, with A2142G, A2142C, and A2143G being the most common.^6^ Most molecular detection of the H. pylori clarithromycin resistance is focused on the above‐mentioned sites. The main specimens used for detection are gastric mucosa, gastric juice, etc. Xiaoli Zheng et al^7^ tested 89 clinical H. pylori strains of Beijing patients. Among them, 12 strains were resistant to clarithromycin. Point mutation of A2143G was detected by PCR‐ RFLP sequence analysis in the 12 resistant strains. Agudo S et al^8^ cultured endoscopic gastric mucosal tissues of 118 Spanish patients and performed E‐test, and 42 resistant strains were detected. Then they detected the common mutation sites of 23 s rRNA gene by PCR‐ RFLP after extracting DNA from drug‐resistant strains and cutting the target fragment by restriction endonuclease BsaI to detect the point mutation of A2143G. The results showed that 85.3% of the resistant strains have point mutation of A2143G. Agudo S et al^9^ detected the H. pylori clarithromycin resistance of H. pylori‐infected children and found point mutations of A2143G, A2142G, and A2142C. In conclusion, with above literature reference, we tested 3 mutations (A2143G, A2142G, and A2142C) at 2 polymorphic loci of H. pylori 23 s rRNA gene as an auxiliary diagnosis of the H. pylori clarithromycin resistance. Gastric mucosa H. pylori tissue specimens from patients were screened based on these common mutations. Resistance genes were then identified using the amplification refractory mutation system combined with quantitative real‐time PCR (ARMS‐PCR) and the gene was sequenced to verify the accuracy of the technique. In addition, a drug susceptibility test was used to determine the minimum inhibitory concentration (MIC) of clarithromycin in H. pylori. The results from the bacterial phenotypic resistance test and the drug resistance loci genotype test were compared to examine the mechanism of drug resistance and to evaluate the consistency of the 2 methods.

## 2 MATERIALS AND METHODS

### 2.1 Samples

In total, 150 in vitro *H. pylori*‐positive culture specimens were harvested from patients at the Peking University First Hospital Digestive Department from June 2015 to June 2016. These patients had unsuccessfully eradicated *H. pylori*: endoscopic examinations, drug sensitivity tests on in vitro cultures, and urea breath tests all showed positive results for H. pylori.

### 2.2 Materials

*Primary instruments and reagents: *LightCycler 480 real‐time fluorescent PCR (Roche Diagnostics, Roche Instrument Center AG, Rotkreuz, Switzerland), Roche LightCycler 480 software version 1.5.1, NanoDrop 2000 ultramicro biological detector (Thermo Fisher), and Taq DNA Polymerase were purchased from Zhuhai baori biotechnology co., Ltd. and dNTPs were purchased from Dalian TaKaRa Co.,Ltd.

#### 2.2.1 Primer probes

Specific upstream ARMS primers, a common downstream primer and probe, and a pair of sequencing primers were designed using the published 23S rRNA gene sequences of the 2142‐2143 loci (Table 1).

**Table 1:** Primer, probe, and sequence information for the *H. pylori* 23S rRNA gene

Name	Sequence
A2142A upstream primer	5’‐CTACCCGCGGCAAGACTGA‐3’
A2142G upstream primer	5’‐CTACCCGCGGCAAGACTGG‐3’
A2142C upstream primer	5’‐CTACCCGCGGCAAGACTGC‐3’
A2143G upstream primer	5’‐CTACCCGCGGCAAGACGTAG‐3’
Common downstream primer	5’‐ATAGGTGGGAGGCTTTGAAGTA‐3’
Common probe	5’‐GACCCCGTGGACCTTTACTACAACT‐3’
Sequencing upstream primer	5’‐GTCAGTCGCAAGATGAAGCGTTG‐3’
Sequencing downstream primer	5’‐CAAGCATTGTCCTGCCTGTGGATAAC‐3’

### 2.3 Methods

#### 2.3.1 *H. pylori* DNA extraction

*H. pylori* DNA was extracted from 150 specimens using the QIAamp DNA Mini Kit (QIAGEN).

#### 2.3.2 Detection by ARMS‐fluorescence PCR

The PCR system contained 10 × PCR buffer, 2 mM MgCl_2_, 0.2 mM dNTPs, 2U Taq enzyme, 0.3 µM common downstream primer, and 0.1 µM common downstream probe. The sample was divided into 3 tubes; then, 3 upstream primers (concentration: 0.03 µM) were added. Finally, the PCR amplification DNA template was added, and the results were analyzed. The reaction conditions were as follows: predenaturation at 94°C for 5 min, denaturation at 94°C for 15 s, and extension at 62°C for 30 s for 40 amplification cycles. Fluorescence signals were collected for each annealing temperature of each cycle.

#### 2.3.3 Sequencing verification

To verify the accuracy of the ARMS‐PCR detection results, 2‐way sequences for the 2142 and 2143 loci of the 23S rRNA gene after sequencing primer amplification were conducted. First, PrimeSTAR® HS (Premix) (Dalian TaKaRa Co., Ltd.) was used for PCR amplification. The reaction system comprised PrimeSTAR HS (Premix, 20 µL), sequencing primer F (10 pmol), sequencing primer R (10 pmol), and the DNA template (100 ng) in a total reaction volume of 50 µL. The reaction conditions were as follows: predenaturation at 94°C for 15 s, degeneration at 98°C for 10 s, extension at 55°C for 15 s, and extension at 72°C extension for 1 min for 30 amplification cycles. Subsequently, the QIAquick PCR Purification Kit was used to purify PCR products, and then a BigDye Terminator v3.1 Cycle Sequencing Kit (QIAGEN) and a 3730 DNA Analyzer (Applied Biosystems, Life Technologies) were used to sequence and analyze the DNA products respectively.

#### 2.3.4 *H. pylori* culture

Gastric mucosa tissues were homogenized with brain heart infusion (BHI), and incubated in Columbia solid medium with 8% defibrinated sheep blood and selective antibiotics (Vancomycin, Polymyxin B, Amphotericin B), in a microaerophilic chamber (5% O2, 10% CO2, and 85% N2; Heal Force Bio‐Meditech Holdings Limited, Shanghai, China). Up to 10 days of observation, colonies were picked up with inoculation loop, and cultured in one or more plates of Columbia solid medium with 8% defibrinated sheep blood, in a microaerophilic chamber (5% O2, 10% CO2, and 85% N2; Heal Force Bio‐Meditech Holdings Limited, Shanghai, China) for 3 days. Well‐growing stains can be used for subsequent experiments, or be collected to BHI with 10% glycerol and stored at −80°C until use.

E‐test drug sensitivity test: The E‐test method (AB Biodisk, Sweden) was used to determine the MIC value of the antibiotic resistance of H. pylori strains to clarithromycin. Freshly cultured *H. pylori *cells were collected on a Columbia agar plate containing 8% goat blood. The *H. pylori *concentration was then adjusted to 3 × 10^8^CFU/ml, which was followed by inoculation on a 9‐cm agar plate in which the L‐rod was coated evenly. After drying, the E‐test strip was added to the culture with the MIC scale facing upward to ensure that the maximum *H. pylori* concentration was near the edge of the plate. The culture conditions were as follows: microaerobic conditions for 72 h at 37°C. The judgment standard of *H. pylori* clarithromycin resistance is an MIC value>1 g/mL.^10^, ^11^

### 2.4 Statistical analysis

SPSS 16.0 software was used for statistical analysis. The counting data were expressed as cases and percentages. A fisher exact test was used to compare the 2 methods. A *P*‐value <.05 was considered statistically significant. Pearson's Correlation Coefficient between 2 methods was calculated. The area under the receiver operating characteristic curve (AUC) analysis was conducted.

## 3 RESULTS

**Figure 1:**
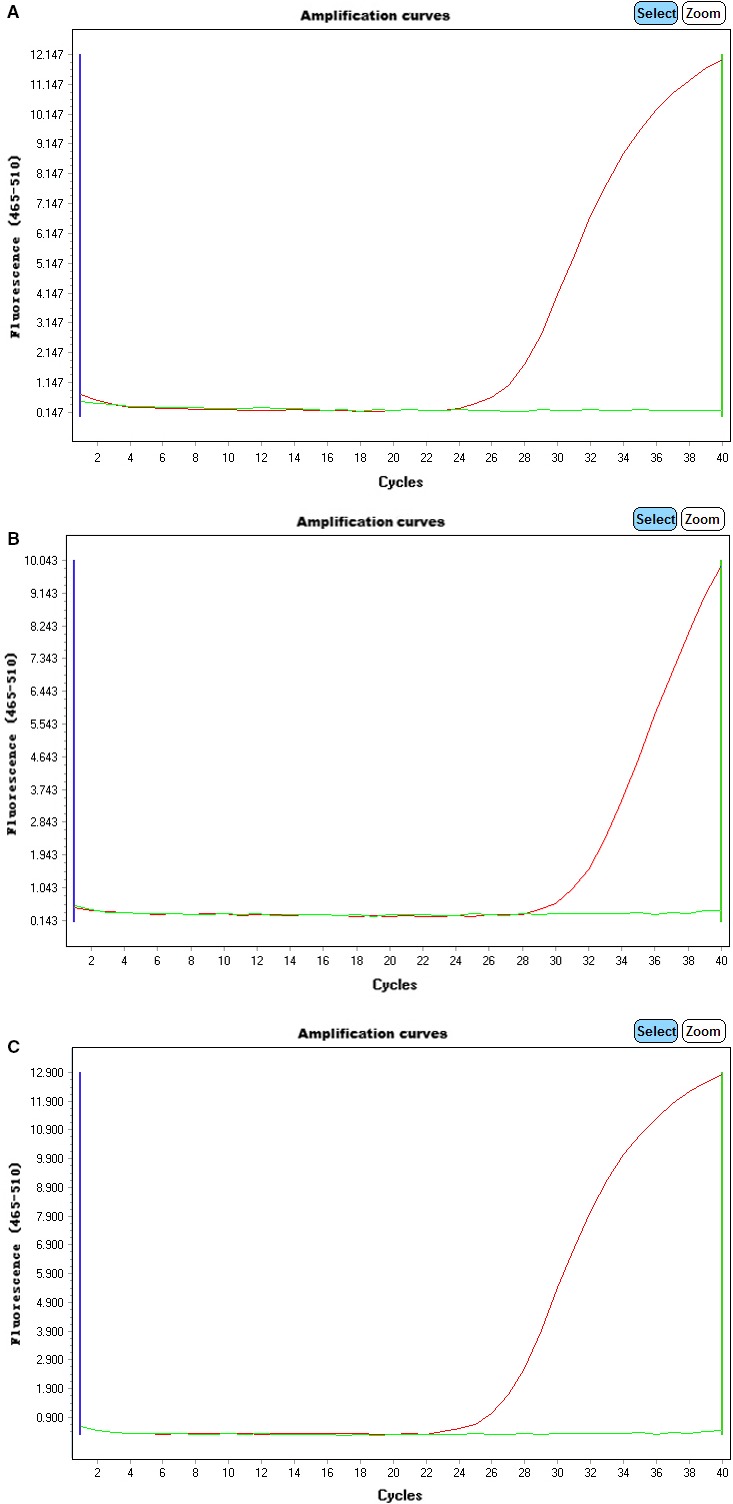
Results of the quantitative real‐time PCR technology based on ARMS Panels (A)‐(C) show mutations at the A2142G, A2142C and A2143G loci, respectively.

**Figure 2:**
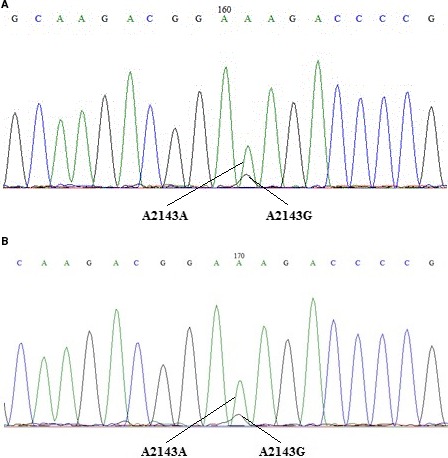
Sequencing results of two specimens which are inconsistent with ARMS‐PCR.

Only one of the 150 gastric mucosa specimens from H. pylori contained an undetected *H. pylori* infection, as determined from the in vitro culture. The other 149 specimens were positive for *H. pylori*. However, the ARMS‐PCR results of 2 cases were inconsistent with the sequencing results. The coincidence rate was 98.7% (147/149) (Figure 1). There were 2 peaks in the mutation sites of the 2 specimens upon further analysis of the sequencing map (Figure 2). This result indicates that a mutation at the locus was present, but the proportion of that mutation was relatively low.

**Figure 3:**
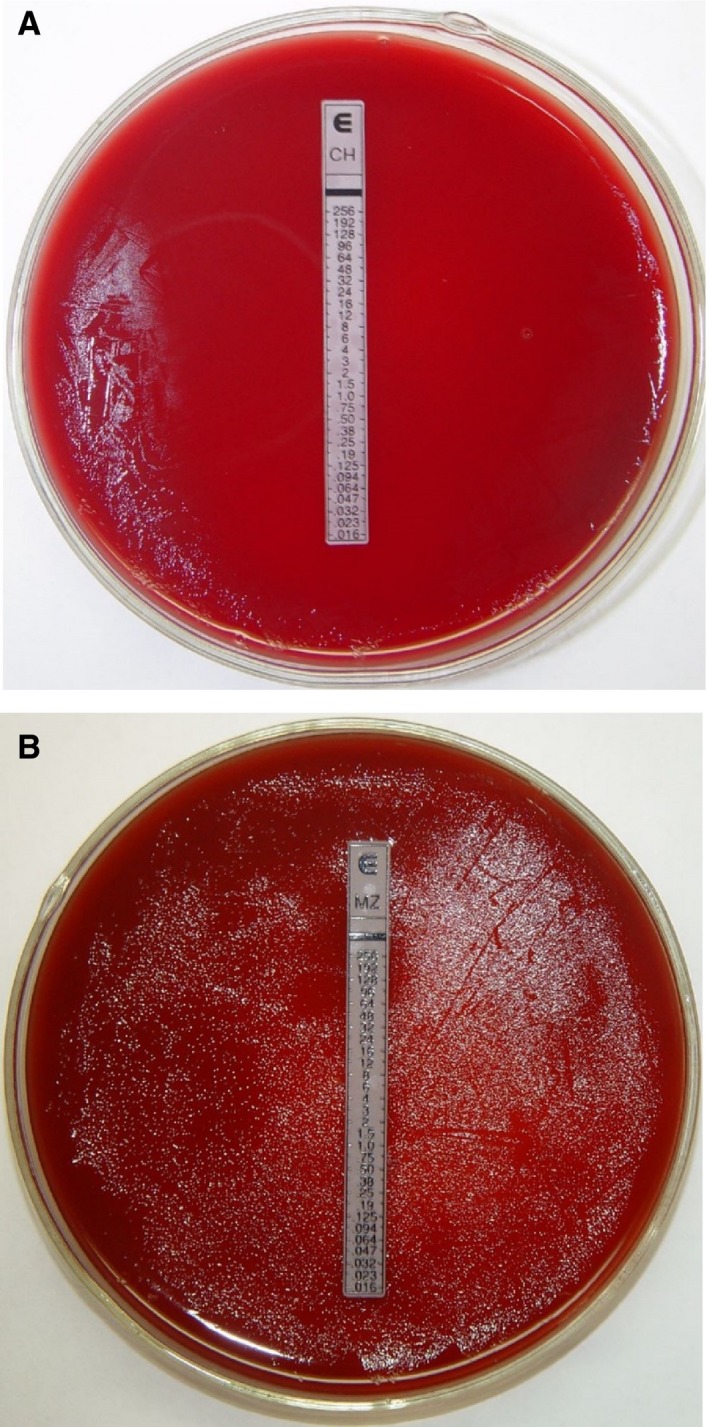
Drug sensitivity test.

A drug sensitivity test for clarithromycin was performed on 149 specimens, which showed that 45 specimens were clarithromycin‐sensitive (30.2%, Figure 3A) and 104 specimens were clarithromycin‐resistant (69.8%, Figure 3B). In addition, ARMS‐PCR was performed on these specimens, and the results showed that 101 specimens were clarithromycin‐resistant. The coincidence rate of the 2 methods was 97.1% (101/104). The clarithromycin resistance rate of the drug sensitivity test (Table 2)was 69.8% (104/149), and the clarithromycin resistance rate of the real‐time fluorescence PCR based on ARMS was 67.8% (101/149). These results were not statistically significant (*P* = .803 > .05) (Table3). Pearson's Correlation Coefficient between 2 methods was 0.95. The area under the receiver operating characteristic curve (AUC) analysis was 0.969.

**Table 2:** Comparison of the drug sensitivity test and ARMS‐fluorescence PCR detection of *H. pylori* clarithromycin resistance gene mutation

		Drug sensitivity test result		Total
		Resistance phenotype	Sensitive phenotype	
ARMS‐fluorescence PCR test result	Mutation	101	0	101
	Wild	3	45	48
	Total	104	45	149

**Table 3:** Results of Fisher's exact test

**Symmetric Measures**						
		Value	Asymp. Std. Error^a^	Approx. T^b^	Approx. Sig.	Exact Sig.
Measure of Agreement	Kappa	0.020	0.054	0.375	0.708	0.803
N of Valid Cases		298				

## 4 DISCUSSION

Currently, the most common treatment used to eradicate H. pylori is antimicrobial agents, which include clarithromycin, metronidazole, amoxicillin, levofloxacin, moxifloxacin, tetracycline, and furazolidone. Clarithromycin is part of a new generation of 14‐ring macrolide antibacterial drugs, is acid‐resistant and dissolves in low‐pH gastric juice. After oral administration, the drug exhibits high bioavailability and few adverse reactions. Both international and domestic consensus recommends the use of clarithromycin for eradication of *H. pylori.* 10, 12 The mechanism of action of clarithromycin occurs via the penetration of bacterial cells with the drug. Once inside the cells, clarithromycin binds to ribosomes, targeting the 23S rRNA at the polypeptide transfer ring. This action results in the inhibition of peptide transferase and affects the ribosome shift process for preventing peptide chain extension, thus inhibiting bacterial protein synthesis and achieving sterilization.^13^

With the extensive use of a series of clarithromycin‐led antibacterial drugs, *H*. pylori clarithromycin resistance has seriously affected the curative effect of treatment regimens containing this drug.^14^, ^15^ The Maastricht V consensus recommendations published online this year suggest that the H. pylori clarithromycin resistance rate was greater than 15%. Furthermore, the recommendations suggested that to use clarithromycin as an eradication treatment, a *H. pylori* clarithromycin resistance test must first be performed.^10^ In this study, only 45 sensitive samples of the 149 cases that failed to eradicate *H. pylori *were found. The resistance rate was 69.8%, indicating that the current *H. pylori* clarithromycin resistance rate has reached a high level. Other studies have shown that individualized *H. pylori* eradication via bacterial drug resistance detection achieves better efficacy than empirical treatment.^16^ Although the resistance rates of *H. pylori *to various antibacterial drugs are not the same worldwide, all regions face the problem of an annually increasing bacterial drug resistance rate, especially for antimicrobial agents including clarithromycin, metronidazole, and quinolones. Therefore, monitoring bacterial resistance and choosing antibacterial drugs in a reasonable manner are significant in assisting in clinical drug use and improving the rate of treatment and eradication.

With the development of experimental techniques in molecular biology, the detection of drug resistance in H. pylori has gradually shifted from traditional bacterial culture and drug‐sensitive tests to molecular biological detection methods.^16^ Gao and Sheng^17^ stated that the application of bacterial‐resistant genotype testing for clinical medication guidance is feasible. They also believe that this method possesses the advantages of traditional drug sensitivity tests. In addition, if it can be applied clinically, this method will rapidly and accurately detect bacterial drug‐resistant gene mutations, thus providing clinical antibacterial drug guidance, and reducing the economic burden on patients. In this study, ARMS‐PCR was first used in the detection of Helicobacter pylori clarithromycin resistance in gastric mucosa. Compared with traditional TaqMan RT‐PCR, ARMS‐PCR effectively reduces nonspecific products such as primer dimer, and improves the specificity and sensitivity of the PCR. Specific ARMS primers were designed according to the 3’‐end mismatch of the principal strand for ARMS‐PCR. These primers were then used to detect 2142 and 2143 mutations on the H. pylori 23S rRNA V domain via fluorescence. Currently, studies on the resistance mechanism of clarithromycin have primarily focused on various sites of the rRNA gene of H. pylori 23S. In 1996, Versalovic et al^18^ first discovered H. pylori resistance to clarithromycin was related to mutations in the V domain of 23S rRNA, and the most common mutation was A2143G. In addition, Taylor^19^ proposed that A2142G possesses a higher MIC than A2143G in the point mutation of the 23S rRNA V domain. The A2142G mutation can simultaneously cause resistance to a variety of drugs including macrolides such as lincomycin and streptomycin. The A2143G mutation is sensitive to streptomycin. H. pylori with A2142G and A2143G mutations exhibit a faster growth rate than that of H. pylori with A2142C, A2143C, or A2142T mutations. Therefore, clarithromycin‐resistant H. pylori contain a larger number of A2142G and A2143G mutations, and, occasionally, H. pylori contain an A2142C mutation. In this study, ARMS‐PCR exhibited a high coincidence rate (98.7%) with the sequencing results. In addition, A2143G mutations were observed in the 2 specimens that did not conform to sequencing map analysis. These results indicate ARMS‐PCR has a higher sensitivity when the mutation rate is low.

In vitro bacterial culture has been the gold standard for the diagnosis of H. pylori infection. This method has also been used to perform drug sensitivity tests for developing individualized clinical treatments. However, this method demands harsh bacterial culture conditions and requires a long time period. The culture conditions affect the results and are not conducive to rapid and large‐scale clinical testing. The ARMS‐PCR method only requires 2 basic processes: gastric mucosal specimen extraction/purification and PCR amplification. Both operations can be completed within 2 h, which is highly suitable for current clinical test requirements. In this study, the coincidence rate between drug‐resistant phenotype detection and drug‐resistant genotype detection was 97.1%. No statistical significance between the 2 detection methods was observed (*P* > .05). Pearson's Correlation Coefficient between 2 methods was 0.95 and the area under the receiver operating characteristic curve (AUC) analysis was 0.969, which indicated that the result of ARMS‐PCR is highly consistent with E‐test MIC drug sensitivity test as golden standard. At a high level, these results indicate that the detection of clarithromycin‐resistant genotypes results in detection of the resistant phenotype. However, in this study, 3 specimens tested as wild‐type by ARMS‐PCR but tested as clarithromycin‐resistant by the drug‐sensitive tests. These results suggest that the clarithromycin‐resistant phenotypes may not be limited to mutations at sites 2142 and 2143 of H. pylori 23S rRNA. These 3 specimens may have mutated at different sites, leading to their drug‐resistant phenotype. In addition, in comparison with the in vitro bacterial culture method, ARMS‐PCR requires the optimization of primers and fluorescent probes. Therefore, inevitably, this method increases the difficulty of the preliminary experimental design and testing costs, which could limit its clinical application.

In conclusion, ARMS‐PCR exhibits a higher sensitivity, a stronger specificity, and faster speed than the traditional culture method. ARMS‐PCR is expected to be suitable for the clinical application of an H. pylori‐resistant mutation detection method. Therefore, this method, as a potential substitution for culture methods when the culture is not easily applicable, could help avoid blindly choosing antimicrobial agents and reducing the failure rate of H. pylori treatment and the cost of treatment.

## CONFLICT OF INTEREST

None Declared.
